# RRM2 Regulates Sensitivity to Sunitinib and PD‐1 Blockade in Renal Cancer by Stabilizing ANXA1 and Activating the AKT Pathway

**DOI:** 10.1002/advs.202100881

**Published:** 2021-07-28

**Authors:** Wei Xiong, Bin Zhang, Haixin Yu, Liang Zhu, Lu Yi, Xin Jin

**Affiliations:** ^1^ Department of Urology The Second Xiangya Hospital Central South University Changsha Hunan 410011 China; ^2^ Uro‐Oncology Institute of Central South University Changsha Hunan 410011 China; ^3^ Cancer center Union Hospital Tongji Medical College Huazhong University of Science and Technology Wuhan 430022 China

**Keywords:** ANXA1, PD‐1 blockade, renal cell carcinomas, RRM2, tyrosine kinase inhibitors

## Abstract

Renal cell carcinoma (RCC) is a malignant tumor of the kidneys. Approximately 70% of RCC cases are clear cell renal cell carcinoma with von Hippel–Lindau (VHL) gene mutation and activation of the vascular endothelial growth factor (VEGF) pathway. Tyrosine kinase inhibitors (TKIs) targeting VEGF have emerged as promising agents for RCC treatment. Apart from primary resistance, acquired resistance to TKIs after initial tumor regression is common in RCC. Recently, immune checkpoint inhibition, including PD‐1/PD‐L1 blockade, alone or in combination with TKIs has improved the overall survival of patients with RCC. Ribonucleotide reductase subunit M2 (RRM2) has been reported in many types of cancer and has been implicated in tumor progression. However, the role of RRM2 in TKIs resistance in RCC remains unclear. In this study, the authors have demonstrated that RRM2 is upregulated in sunitinib‐resistant RCC cells and patient tissues. They also find that RRM2 stabilizes ANXA1 and activates the AKT pathway independent of its ribonucleotide reductase activity, promoting sunitinib resistance in RCC. Moreover, RRM2 regulated antitumor immune responses, and knockdown of RRM2 enhance the anti‐tumor efficiency of PD‐1 blockade in renal cancer. Collectively, these results suggest that aberrantly expressed RRM2 may be a promising therapeutic target for RCC.

## Graphical abstract

RRM2 overexpression plays a key role in sunitinib resistance in patients with renal cell carcinoma and that RRM2 competes with UBE3A to prevent ANXA1 degradation. The up‐regulated ANXA1 activates AKT signaling pathway to regulate the sensitivity to sunitinib and PD‐1 blockade in renal cancer cells.

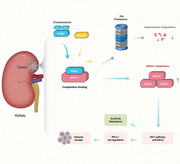

## 1 Introduction

Renal cell carcinoma (RCC) is a heterogeneous malignant tumor of the kidneys, accounting for 2–3% of all types of cancer worldwide.^[^ ^1^ ^]^ The incidence of RCC has been increasing in recent decades.^[^ ^2^ ^]^ The prognosis of RCC varies depending on the stage of diagnosis.^[^ ^2^ ^]^ For the reason that the symptoms and signs of RCC are non‐specific, ≈25% of patients are diagnosed with advanced or metastatic disease by incidental abdominal imaging.^[^ ^3^ ^]^ Systemic therapy is the only treatment option for patients with metastatic RCC.

Approximately 70% of RCC cases are clear cell renal cell carcinoma (ccRCC). ccRCCs are characterized by mutations in the von Hippel–Lindau (VHL) gene and activation of hypoxia signaling due to dysregulation of proteasomal degradation of hypoxia‐inducible factor (HIF).^[^ ^4^ ^]^ HIF overexpression modulates the metabolism of renal cancer cells and upregulates genes associated with angiogenesis.^[^ ^5^ ^]^ Tyrosine kinase inhibitors (TKIs) targeting vascular endothelial growth factor (VEGF) have emerged as promising agents for the treatment of RCC.^[^ ^6^ ^]^ In 2005, two TKIs (sorafenib and sunitinib) were approved for the treatment of metastatic RCC (mRCC),^[^ ^7^ ^]^ the use of which has substantially prolonged the survival of patients with mRCC.^[^ ^7^ ^]^ However, some RCCs progress despite VEGF signaling inhibition and acquired resistance to TKIs is common.^[^ ^8^ ^]^

Immune checkpoint inhibition, including PD‐1/PD‐L1 blockade, greatly improves the prognosis of patients with mRCC.^[^ ^9^ ^]^ HIF has been shown to upregulate PD‐L1 on tumor cells; similarly, VEGF upregulates PD‐1 on immune cells, promoting T cell exhaustion in the RCC microenvironment.^[^ ^10^ ^]^ Thus, patients with mRCC may benefit from the combination of TKIs with PD‐1/PD‐L1 blockade.^[^ ^11^ ^]^ Recent findings from clinical trials suggest that the combination of TKIs with PD‐1/PD‐L1 inhibitors may improve the overall survival of patients with RCC.^[^ ^10^ ^]^

Ribonucleotide reductase subunit M2 (RRM2) is a ribonucleotide reductase (RNR) subunit that catalyzes the synthesis of deoxyribonucleotides.^[^ ^12^ ^]^ RRM2 overexpression has been reported in many types of cancer and has been implicated in tumor progression.^[^ ^13^ ^]^ Additionally, microRNA (miR)‐99a‐3p targeting RRM2 mRNA is often downregulated in sunitinib‐resistant RCC cells,^[^ ^14^ ^]^ suggesting that RRM2 may be overexpressed in sunitinib‐resistant RCC cells. However, the role of RRM2 in sunitinib resistance in RCC remains unclear. In this study, we have demonstrated that RRM2 is upregulated in sunitinib‐resistant RCC cells and patient tissues. We also found that RRM2 stabilized ANXA1 and activated the AKT pathway independent of its ribonucleotide reductase activity, promoting sunitinib resistance in RCC. Furthermore, we have shown that knockdown of RRM2 enhanced the anti‐tumor efficiency of PD‐1 blockade in RCC. From our data, it can be observed that RRM2 may be a promising therapeutic target for RCC.

## 2 Results and Discussion

### 2.1 RRM2 Overexpression Regulates Sunitinib Sensitivity in Renal Cancer

**Figure 1:**
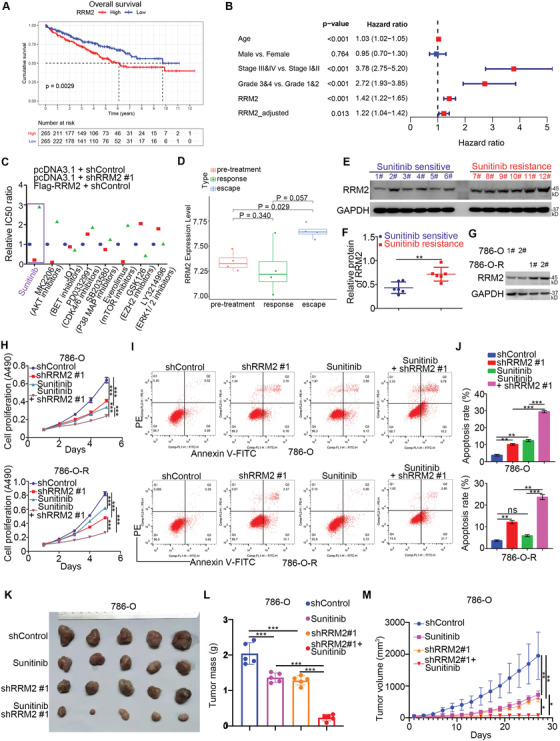
RRM2 overexpression regulates sunitinib sensitivity in renal cancer. A) The overall survival rate in the high/low RRM2 group was analyzed using the TCGA dataset, the *p* value is as indicated. The median RRM2 expression level was used as the cutoff value. B) Univariate Cox regression analysis and multivariate Cox regression analysis adjusted for age, sex, tumor stage, and tumor grade. C) 786‐O cells were infected with lentivirus‐expressing shControl or shRRM2. Seventy‐two hours later, the shRRM2 group was transfected with RRM2 plasmids for 24 h. The cells were treated with a series of concentrations of chemicals as indicated. Cell viability was evaluated using the MTS assay (*n* = 3). The IC50 ratio between shControl versus shControl, shControl versus shRRM2, and Flag‐RRM2 versus shcontrol are shown in panel (C). For the sunitinib treatment group, three replicates were used, and one‐way ANOVA followed Turkey's multiple comparisons post hoc test for the statistical analysis. The *p* values for shControl versus shRRM2 were 0.0001, and the *p* values for Flag‐RRM2 versus shControl were less than 0.0001. D) Expression levels of RRM2 during sunitinib pre‐treatment (*n* = 4), response (*n* = 4), and resistance (escape) (*n* = 4) phases. One‐way ANOVA followed by Turkey's multiple comparisons post hoc test was applied for the statistical analysis. The *p* values are shown in the figure. E,F) The protein level of RRM2 from renal cell carcinoma patients with (*n* = 6) or without (*n* = 6) sunitinib resistance was examined by Western blotting analysis (E), and the protein level of RRM2 was quantified by Image J software (F). Student's *t* test was used to determine statistical significance. **, *p* < 0.01. G) Western blotting analysis of the whole cell lysates (WCL) of 786‐O and 786‐O R cells. H–J) 786‐O, and 786‐O R cells were infected with lentivirus‐expressing shControl or shRRM2. Seventy‐two hours later, 786‐O cells were treated with 10 µm sunitinib and 786‐O R with 30 µm sunitinib for 24 h. These cells were harvested for MTS assay (H) (*n* = 3) and Annexin V/PE staining analysis (I and J) (*n* = 2). Data presented as mean ± SD. One‐way ANOVA followed by Turkey's multiple comparisons post hoc test was applied for the statistical analysis. Ns, not significant; *, *p* < 0.05; **, *p* < 0.01; ***, *p* < 0.001. K–M) 786‐O cells were infected with lentivirus‐expressing shControl or shRRM2. After 72 h of infection and puromycin selection, cells were subcutaneously injected into nude mice for the xenograft assay and treated with or without sunitinib (20 mg kg^−1^). The image of tumor is shown in panel (K). The tumor mass was demonstrated as in panel (L). The tumor growth curve was as indicated in panel (M). Data are presented as mean ± SD with five replicates. One‐way ANOVA followed by Turkey's multiple comparisons post hoc test was applied for the statistical analysis. *, *p* < 0.05; **, *p* < 0.01; ***, *p* < 0.001.

RRM2 has been identified as a poor prognostic marker in several types of cancer.^[^ ^16^ ^]^ In this study, we found that RRM2 was dramatically upregulated in RCC and other types of cancer in the TCGA database (Figure S1A,B, Supporting Information). Kaplan–Meier survival analyses showed that RRM2 upregulation was associated with poor overall survival in patients with RCC (**Figure** **1**A); this association was true even after adjusting for various confounding factors (e.g., age, sex, stage, and grade) in multivariate cox regression analysis (Figure 1B). Additionally, high RRM2 expression levels were strongly associated with advanced disease stage and high tumor grade in patients with RCC (Figure S1C,D, Supporting Information), suggesting that RRM2 is a poor prognostic factor in RCC.

We then examined the oncogenic function of RRM2 in RCC by silencing its expression in 786‐O and A498 renal cancer cells using shRNAs (Figure S1E, Supporting Information). The proliferation of renal cancer cells and the growth of renal cancer xenografts in nude mice were significantly decreased after RRM2 knockdown (Figure S1F,H–J, Supporting Information). In contrast, RRM2 overexpression enhanced the proliferation of renal cancer cells (Figure S1G, Supporting Information), suggesting that RRM2 plays the role of growth promotion in RCC.

We conducted a drug‐screening assay in 786‐O cells, which has a relatively higher RRM2 expression level compared with other RCC cell lines (Figure S1K, Supporting Information), with RRM2 knockdown or overexpression. The IC50 values of sunitinib, the AKT inhibitor MK2206, and the mTOR inhibitor everolimus in RRM2 knockdown cells were lower than those in the control group (Figure 1C); in contrast, RRM2‐overexpressing cells exhibited higher IC50 values for sunitinib, MK2206, and everolimus (Figure 1C). Currently, sunitinib is a multi‐targeted TKI and standard first‐line therapy for patients with advanced RCC. TKI resistance has become a major problem in prolonging the survival time of advanced RCC. We performed tight clustering analysis to show that angiogenesis‐related genes and RRM2 were upregulated in TKI resistance (GSE76068) (Figure 1D and Figure S2A, Supporting Information). Consistently, there was a higher RRM2 protein level in tissues of patients with RCC with sunitinib resistance compared to that in patients without sunitinib resistance (Figure 1E,F). In addition, we constructed sunitinib‐resistant 786‐O cells (786‐O R) as previously described.^[^ ^15^ ^]^ Similarly, we have shown that RRM2 protein levels in 786‐O R cells (derived from two clones) were higher than those in 786‐O cells (Figure 1G). Hence, these data suggest that RRM2 may affect the sensitivity of RCC to sunitinib. To confirm this, we silenced RRM2 in 786‐O and 786‐O R cells treated with or without sunitinib. MTS and Annexin V/PE assays indicated that RRM2 silencing increased sunitinib sensitivity and promoted apoptosis in both sunitinib‐sensitive and sunitinib‐resistant renal cancer cells (Figure 1H–J). Moreover, RRM2 silencing augmented the antitumor effects of sunitinib in vivo (Figure 1K–M). Therefore, these data suggest that RRM2 overexpression promotes sunitinib resistance in RCC.

### 2.2 RRM2 Activates the AKT Pathway Independent of its Enzymatic Activity

**Figure 2:**
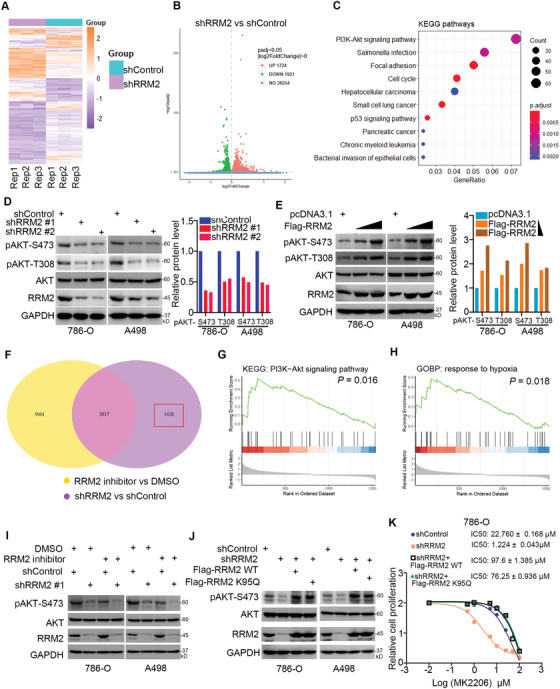
RRM2 activates the AKT pathway independent of its enzymatic activity. A–C) 786‐O cells were infected with the indicated constructs for 72 h. Cells were subjected to RNA‐seq analysis (A and B) and subsequent KEGG pathway enrichment (C). D) 786‐O and A498 cells were infected with the indicated constructs for 72 h. Cells were harvested for Western blotting analysis. The protein level of pAKT‐S473 was quantified by Image J software. E) 786‐O and A498 cells were transfected with Flag‐RRM2 plasmids (5 and 15 ng) for 24 h. Cells were harvested for Western blotting analysis. The protein level of pAKT‐S473 was quantified by Image J software. F) 786‐O cells were treated with or without RRM2 inhibitors (COH29, 15 µm) for 24 h. Cells were subjected to RNA‐seq analysis. The Venn map showed that the genes changed after knockdown of RRM2 or treated with RRM2 inhibitors. G,H) KEGG enrichment analysis (G) and GOBP enrichment analysis (H) of 1628 genes in the RRM2 knockdown group independent of RRM2 inhibitor. *p* values are indicated in the figure. I) 786‐O and A498 cells were infected with the indicated constructs for 72 h. These cells were treated with or without 20 µm COH29 for another 24 h. Cells were harvested for Western blotting analysis. J) 786‐O and A498 cells were infected with the indicated constructs for 72 h. These cells were transfected with the indicated plasmids for 24 h. Cells were harvested for Western blotting analysis. K) 786‐O cells were pretreated with 20 µm COH29. These cells were infected with shControl or shRRM2 for 48 h. Then, the shRRM2 group cells were transfected with or without Flag‐RRM2 for 24 h. These cells were treated with a series of concentrations of sunitinib for 24 h. Cells viability was measured by MTS assay.

To elucidate the mechanisms by which RRM2 regulates sunitinib sensitivity, we performed RNA‐seq analysis in 786‐O cells after RRM2 silencing (**Figure** **2**A,B). KEGG pathway enrichment analysis indicated that RRM2 silencing inhibited the PI3K/AKT signaling pathway (Figure 2C). Moreover, RRM2 knockdown decreased AKT phosphorylation levels and increased the antitumor effects of MK2206 and everolimus in renal cancer cells (Figure 2D; Figure S2B,C, Supporting Information). Contrarily, RRM2 overexpression activated the AKT pathway and reduced the sensitivity of renal cancer cells to MK2206 and everolimus (Figure 2E; Figure S2D,E, Supporting Information). PI3K/AKT signaling has been implicated in sunitinib resistance in RCC. MK2206 pretreatment of 786‐O cells diminished the ability of RRM2 knockdown or overexpression to affect the IC50 values of sunitinib, suggesting that RRM2 promotes sunitinib resistance by activating AKT signaling (Figure S2F, Supporting Information).

We then assessed to find if the ability of RRM2 to regulate AKT signaling was dependent on its enzymatic activity. RNA‐seq analysis of 786‐O cells treated with or without RRM2 inhibitors revealed that 1628 genes after RRM2 knockdown were independent of the enzymatic activity of RRM2 (Figure 2F). KEGG pathway enrichment analysis showed that these 1628 genes were closely related to PI3K/AKT pathway activation (Figure 2G). Interestingly, enrichment analysis of the GO biological process indicated that these 1628 genes were associated with hypoxia (Figure 2H). Furthermore, we have shown that RRM2 silencing decreased the phosphorylation of AKT and increased the sensitivity of renal cancer cells pretreated with the RRM2 inhibitor COH29 (Figure 2I; Figure S2G, Supporting Information). Expression of the K95Q mutant RRM2 considerably decreased the ribonucleotide reductase activity of RRM2,^[^ ^17^ ^]^ increased AKT phosphorylation levels, and reduced sensitivity to AKT inhibitors in renal cancer cells (Figure 2J,K). Hence, these results suggest that, at least partially, RRM2 modulates AKT activation independent of its ribonucleotide reductase activity.

### 2.3 RRM2 Binds to ANXA1 to Activate AKT Signaling in Renal Cancer Cells

**Figure 3:**
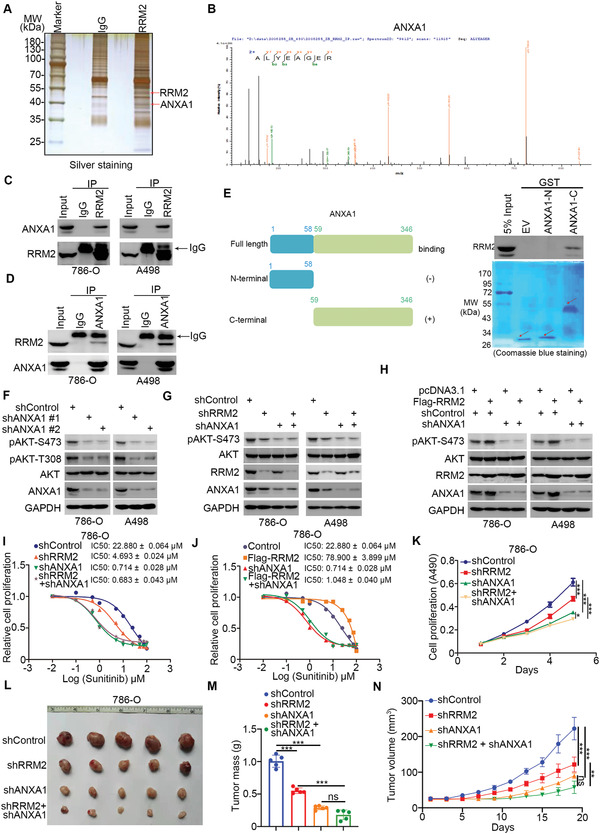
RRM2 binds to ANXA1 to activate AKT signaling in renal cancer cells. A,B) The WCL of 786‐O cells were subjected to silver staining (A) and mass spectrometry with IgG and RRM2 antibodies (A). The peptide of ANXA1 pulled down by RRM2 is as indicated in panel (B). C,D) Western blotting analysis of the WCL of 786‐O and A498 cells. E) A schematic diagram depicting the domain of ANXA1. Western blotting analysis of RRM2 GST‐pulled down by ANXA1 recombinant. F) 786‐O and A498 cells were infected with indicated constructs for 72 h. Cells were harvested for Western blotting analysis. G) 786‐O and A498 cells were infected with indicated constructs for 72 h. Cells were harvested for Western blotting analysis. H) 786‐O and A498 cells were infected with indicated constructs for 72 h. Then, these cells were transfected with indicated plasmids for 24 h. Cells were harvested for Western blotting analysis. I) 786‐O and A498 cells were infected with indicated constructs for 72 h. Cells were treated with a series of concentration of sunitinib for 24 h. Cells viability was measured by MTS assay. J) 786‐O and A498 cells were infected with indicated constructs for 72 h. Then, these cells were transfected with indicated plasmids for 24 h. Cells were treated with a series of concentration of sunitinib for 24 h. Cells viability was measured by MTS assay. K–N) 786‐O cells were infected with indicated constructs for 72 h. Cells were harvested for MTS assay and xenografts assay. The image of tumor is shown in panel (L). The tumor mass was demonstrated as in panel (M). The tumor growth curve is indicated in panel (N). Data are presented as mean ± SD with five replicates. One‐way ANOVA followed by Turkey's multiple comparisons post hoc test was applied for the statistical analysis. Ns, not significant; **, *p* < 0.01; ***, *p* < 0.001.

To explore the mechanisms by which RRM2 activates AKT in renal cancer cells, we employed mass spectrometry to identify the potential binding partners of RRM2 (**Figure** **3**A). We identified ANXA1 as the most significant binding partner of RRM2 with the highest log2(RRM2‐immunoprecipitation/RRM2‐IgG) ratio in this mass spectrometry assay (Figure 3B; Table S3, Supporting Information). It has been well documented that ANXA1 is responsible for the activation of the PI3K/AKT signaling pathway in cancer cells by interacting with the formyl peptide receptors (FPRs) FPR1 and FPR2,^[^ ^18^, ^19^ ^]^ thus, we were curious to find RRM2 regulated the activation of the AKT signaling axis through ANXA1. Co‐immunoprecipitation (co‐IP) confirmed that RRM2 interacted with ANXA1 in both 786‐O and A498 cells (Figure 3C,D). To identify the region of ANXA1 that mediates its interaction with RRM2, we constructed an ANXA1 recombinant protein. GST pull‐down indicated that RRM2 binds to the C‐terminus of ANXA1 (Figure 3E). Consistent with the role of ANXA1 in AKT pathway activation,^[^ ^20^ ^]^ ANXA1 silencing decreased AKT phosphorylation levels in 786‐O and A498 cells (Figure 3F), but overexpression of ANXA1 resulted in the upregulation of AKT phosphorylation at T308 and S473 sites in 786‐O and A498 cells (Figure S2H, Supporting Information). Intriguingly, ANXA1 silencing attenuated the ability of RRM2 knockdown or overexpression to alter AKT phosphorylation levels and sensitivity to MK2206 in renal cancer cells (Figure 3G–J). Moreover, knockdown of ANXA1 or RRM2 alone suppressed tumor growth in mice. Notably, simultaneous knockdown of ANXA1 and RRM2 was more potent than ANXA1 silencing alone in suppressing tumor growth (Figure 3K–N). Therefore, these results suggest that ANXA1 is a critical mediator of RRM2's ability to activate AKT in renal cancer.

### 2.4 RRM2 Competes with UBE3A to Stabilize ANXA1 in Renal Cancer Cells

**Figure 4:**
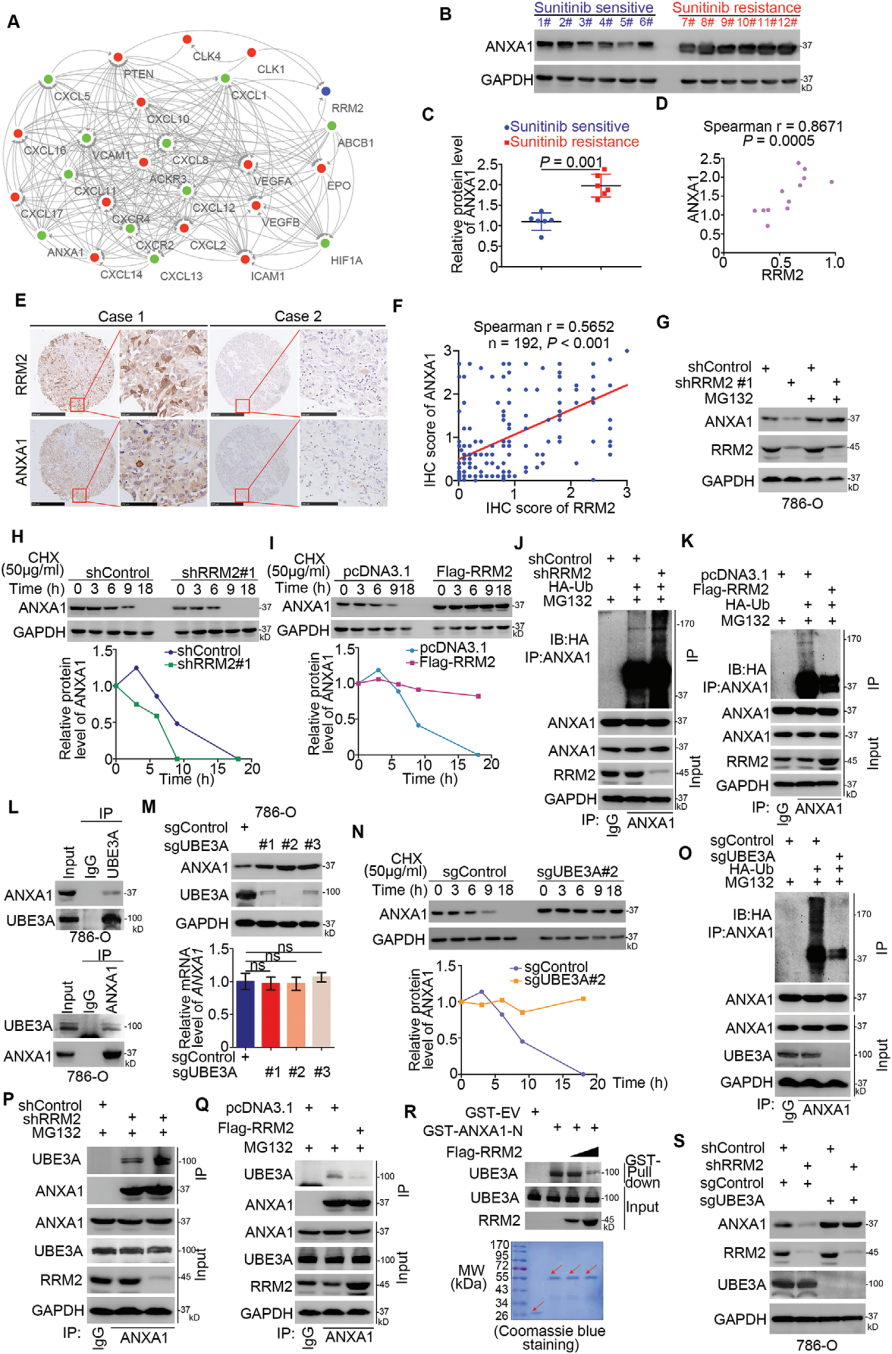
RRM2 competes with UBE3A to stabilize ANXA1 in renal cancer cells. A) PPI networks of RRM2, ANXA1, and 24 genes involved in sunitinib response and resistance. SKI was not included because it had no interactions with other proteins. The blue node represents RRM2. Nodes in red represent upregulated DEGs in the RRM2 high expression group versus the low expression group in TCGA‐KIRC. Nodes in green represent downregulated DEGs in the RRM2 high expression group versus the low expression group in TCGA‐KIRC. Arrows indicate the direction from source nodes to target nodes. B,C) The protein level of ANXA1 from renal cell carcinoma patients with (*n* = 6) or without (*n* = 6) sunitinib resistance was examined by Western blotting analysis (B), the protein level of RRM2 was quantified by Image J software (C). Student's *t* test was used to determine the statistical significance. **, *p* = 0.001. D) The correlation between the protein levels of RRM2 and ANXA1 in renal cell carcinoma patients with or without sunitinib resistance, the *p* value is as indicated. E,F) The tissue microarray of renal cancer stained with RRM2 and ANXA1, respectively. The typical IHC images of stained with RRM2 and ANXA1 are shown in panel (E). The correlation of these two proteins is shown in panel (F), the *p* value was indicated in the figure. G) 786‐O cells were infected with indicated constructs for 72 h. Cells were harvested for Western blotting analysis before treatment with or without MG132. H) 786‐O cells were infected with indicated shRNAs. After 72 h, cells were treated with Cycloheximide (CHX) and cells were collected for Western Blotting analysis at different time points. I) 786‐O cells were transfected with indicated plasmids. After 24 h, cells were treated with CHX and cells were collected for Western Blotting analysis at different time points. J) 786‐O cells were infected with indicated constructs. Seventy‐two hours post‐infection, cells were collected for Western Blotting analysis after being treated with MG132 for 8 h. K) 786‐O cells were transfected with indicated constructs. After 24 h, cells were collected for Western Blotting analysis after being treated with MG132 for 8 h. L) Western blotting analysis of the WCL of 786‐O cells. M) The 786‐O cells were transfected with indicated constructs. Fourteen days after selection, these cells were harvested for Western blotting and RT‐qPCR analysis. Data are presented as mean ± SD with three replicates. Ns, not significant. N) The 786‐O cells transfected with or without sgUBE3A were treated with CHX and cells were collected for Western Blotting analysis at different time points. O) The 786‐O cells transfected with or without sgUBE3A were transfected with indicated constructs. After 24 h, cells were collected for Western Blotting analysis after being treated with MG132 for 8 h. P) 786‐O cells were infected with indicated constructs. 72 h post‐infection, cells were collected for Western Blotting analysis after being treated with MG132 for 8 h. Q) 786‐O cells were transfected with indicated constructs. After 24 h, cells were collected for Western Blotting analysis after being treated with MG132 for 8 h. R) Western Blotting analysis of in vitro expressed UBE3A GST‐Pulled down by ANXA1. S) Western Blotting analysis of the WCL of cells transfected with indicated constructs.

It was worth noting that RRM2 silencing decreased the protein level of ANXA1 but RRM2 overexpression upregulated ANXA1 protein levels in renal cancer cells (Figure 3G,H). Protein–protein interaction (PPI) network analyses revealed that, among the proteins involved in sunitinib resistance, there was a certain connection between RRM2 and ANXA1 (**Figure** **4**A). Moreover, we found that the protein levels of ANXA1 in RCC tissues with sunitinib resistance were significantly higher than those in sunitinib‐sensitive tissues (Figure 4B,C). Interestingly, ANXA1 protein levels were positively correlated with RRM2 levels in RCC tissues (*n* = 12, Spearman *r* = 0.867, *p* = 0.0005) and in RCC tissue microarrays (*n* = 192, Spearman *r* = 0.5652, *p* < 0.001). Thus, we hypothesized that RRM2 might affect ANXA1 in renal cancer cells; to test this hypothesis, RRM2 was knocked down in renal cancer cells, and the knockdown of RRM2 decreased the protein levels of ANXA1, but had no effect on the mRNA levels of ANXA1 (Figure 4G; Figure S3A, Supporting Information). The 26S proteasome inhibitor MG132 attenuated ANXA1 downregulation upon RRM2 silencing (Figure 4G). Furthermore, RRM2 knockdown shortened the half‐life of ANXA1 and increased ANXA1 ubiquitination levels (Figure 4H,J). Conversely, RRM2 overexpression prolonged the half‐life of ANXA1 and reduced ANXA1 ubiquitination levels (Figure 4I,K). The E3 ligase UBE3A (E6AP) has been previously shown to bind to the C‐terminal domain of ANXA1, causing ANXA1 degradation.^[^ ^21^ ^]^ Co‐IP in 786‐O cells confirmed that ANXA1 interacted with UBE3A (Figure 4L). UBE3A knockout increased the protein but not the mRNA levels of ANXA1 in 786‐O cells (Figure 4M).

Consistent with our previous findings, UBE3A knockout prolonged the half‐life and decreased the ubiquitination levels of ANXA1 in 786‐O cells (Figure 4N,O), demonstrating that ANXA1 is also a bona fide substrate of UBE3A in 786‐O cells. Interestingly, GST pull‐down analysis showed that the C‐terminal domain of ANXA1 interacted with RRM2 (Figure 3E). We also found that RRM2 competed with UBE3A to bind to the C‐terminal domain of ANXA1 in vivo and in vitro (Figure 4P–R). Consistently, RRM2 silencing failed to downregulate ANXA1 in UBE3A‐knockout 786‐O cells (Figure 4S). Hence, these data suggest that, by competing with UBE3A, RRM2 binds to and stabilizes ANXA1 in renal cancer cells.

### 2.5 RRM2 Upregulates PD‐L1 in Renal Cancer Cells

**Figure 5:**
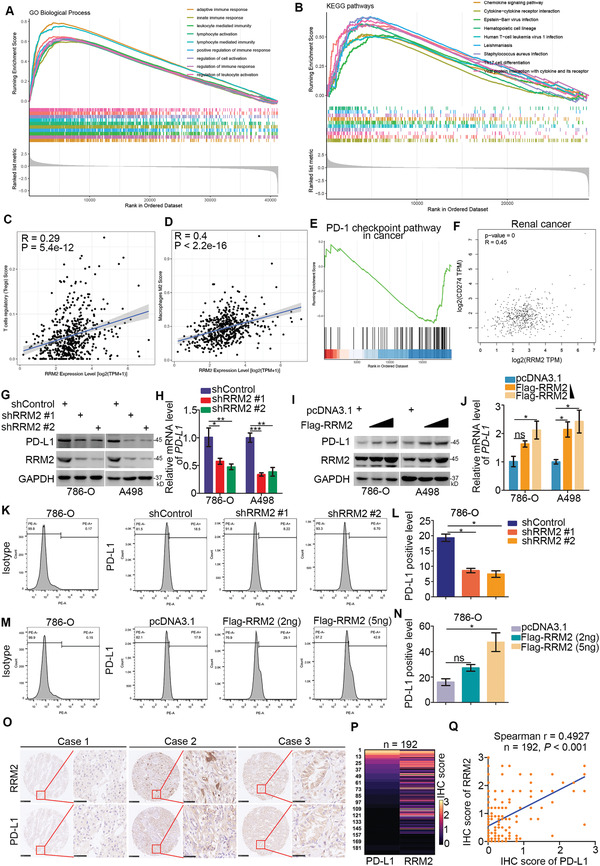
RRM2 upregulates PD‐L1 in renal cancer cells. A,B) GSEA analysis for RRM2 by GO biological process and KEGG enrichment in the TCGA‐KIRC (kidney renal clear cell carcinoma) dataset. C,D) The infiltration level of Treg or macrophages M2 cells based on the expression of RRM2 in renal cancer patients. E) The GSEA analysis of 1682 genes regulated by RRM2 knockdown, but not RRM2 inhibitors in 786‐O cells. F) The correlation between the mRNA levels of RRM2 and PD‐L1 was analyzed by the GEPIA web tool (http://gepia.cancer‐pku.cn/). G, H, K,L) 786‐O and A498 cells were infected with the indicated constructs for 72 h. Cells were harvested for the Western blotting (G), RT‐qPCR (H) (*n* = 3), and FACS analysis (K and L) (*n* = 2). Data are presented as mean ± SD. One‐way ANOVA followed by Turkey's multiple comparisons post hoc test was applied for the statistical analysis. The *p* values were shown in the figure. Ns, not significant; *, *p* < 0.05; **, *p* < 0.01; ***, *p* < 0.001. I,J,M,N) 786‐O and A498 cells were transfected with the indicated constructs for 24 h. Cells were harvested for the Western blotting (I), RT‐qPCR (J) (*n* = 3), and FACS analysis (M and N) (*n* = 2). Data are presented as mean ± SD. One‐way ANOVA followed by Turkey's multiple comparisons post hoc test was applied for the statistical analysis. Ns, not significant; *, *p* < 0.05. O–Q) The tissue microarray of renal cancer was stained with RRM2 and PD‐L1. Typical IHC images stained with RRM2 and PD‐L1 are shown in panel (O). The expression levels of RRM2 and PD‐L1 are shown in the heatmap (P). The correlation of these two proteins is shown in panel (Q), the *p* value was indicated in the figure.

Gene set enrichment analysis (GSEA) analysis revealed that RRM2 was closely associated with several immune‐related pathways in the kidney renal clear cell carcinoma (KIRC) TCGA dataset (**Figure** **5**A,B). Moreover, RRM2 levels were positively correlated with the number of immunosuppressive regulatory T cells (Tregs) and M2 macrophages in the TCGA‐KIRC dataset (Figure 5C,D). GSEA analysis of our RNA‐seq data indicated that RRM2 might modulate the PD‐1 pathway in 786‐O cells (Figure 5E). Intriguingly, analyses using the GEPIA web tool demonstrated a significant association between RRM2 and PD‐L1 levels in renal cancer, bladder cancer, breast cancer, and prostate cancer tissues (Figure 5F; Figure S3B, Supporting Information). To confirm the role of RRM2 in PD‐L1 expression in RCC, we evaluated PD‐L1 expression levels after RRM2 knockdown. RRM2 silencing decreased the mRNA and protein levels of PD‐L1 in 786‐O and A498 cells (Figure 5G,H,K,L). Consistently, RRM2 overexpression increased PD‐L1 expression levels in renal cancer cells (Figure 5I,J,M,N). We also analyzed the expression levels of RRM2 and PD‐L1 in RCC tissue microarrays by immunohistochemistry (Figure 5O). RRM2 protein levels were positively correlated with PD‐L1 expression in RCC tissues (*n* = 192, Spearman *r* = 0.4927, *p* < 0.001), suggesting that RRM2 positively regulates PD‐L1 expression in RCC.

### 2.6 RRM2 Modulates Immune Responses in RCC by Regulating the ANXA1/AKT Signaling Axis

**Figure 6:**
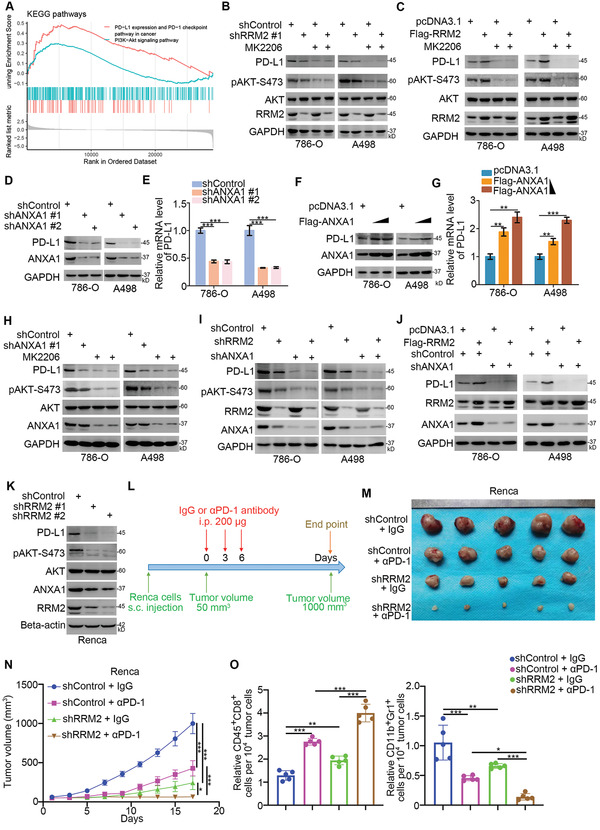
RRM2 modulates immune responses in RCC by regulating the ANXA1/AKT signaling axis. A) KEGG enrichment analysis of RRM2 in TCGA datasets. B) 786‐O and A498 cells were infected with the indicated constructs for 72 h. Cells were treated with or without MK2206 (50 µm) for 24 h and harvested for Western blotting analysis. C) 786‐O and A498 cells were transfected with the indicated constructs for 24 h. Cells were treated with or without MK2206 (50 µm) for 24 h and harvested for Western blotting analysis. D,E) 786‐O and A498 cells were infected with indicated constructs for 72 h. Cells were harvested for the Western blotting (D) and RT‐qPCR (E) assay (*n* = 3). Data are presented as mean ± SD. One‐way ANOVA followed by Turkey's multiple comparisons post hoc test was applied for the statistical analysis. ***, *p* < 0.001. F,G) 786‐O and A498 cells were transfected with different dose of ANXA1 plasmids (2 and 6 ng) for 24 h. Cells were harvested for the Western blotting (F) and RT‐qPCR (G) analysis (*n* = 3). Data are presented as mean ± SD. One‐way ANOVA followed by Turkey's multiple comparisons post hoc test was applied for the statistical analysis. **, *p* < 0.01; ***, *p* < 0.001. H) 786‐O and A498 cells were infected with indicated constructs for 72 h. Cells were treated with or without MK2206 (50 µm) for 24 h and harvested for Western blotting analysis. I) 786‐O and A498 cells were infected with indicated constructs for 72 h. Cells were harvested for Western blotting analysis. J) 786‐O and A498 cells were infected with indicated constructs. 72 h post‐infection, cells were transfected with the indicated plasmids for another 24 h. Cells were harvested for Western blotting analysis. K–O) Renca cells were infected with lentivirus vectors expressing control or RRM2 shRNAs. After 72 h, cells were harvested for Western blotting analysis (K). These cells were infected with sh‐Control or sh‐RRM2 and subcutaneously injected into the right dorsal flank of C57BL/6 mice. Mice with subcutaneous Renca tumors (*n* = 5/group) were treated with anti‐PD‐1 (200 µg) or nonspecific IgG three times as shown in the schematic diagram (L). The image of tumor is shown in panel (M). The tumor growth curve was demonstrated as in panel (N). At the end of treatment, the tumors excised from the mice were dissociated and tumor cells were harvested for flow cytometry analysis to detect the number of TILs. All data are shown as means ± SD (*n* = 5). One‐way ANOVA followed by Turkey's multiple comparisons post hoc test was applied for the statistical analysis. *, *p* < 0.05; **, *p* < 0.01; ***, *p* < 0.001.

It has been documented that the PI3K‐AKT pathway activates NF‐*κ*B to transcriptionally increase PD‐L1 expression.^[^ ^22^ ^]^ In our previous study, we have also mentioned that FGD1 increased PD‐L1 expression through the PTEN/PI3K/AKT/NF‐*κ*B signaling axis.^[^ ^23^ ^]^ Moreover, treatment with a PI3K inhibitor was also observed to reduce PD‐L1 expression in melanoma cells.^[^ ^24^ ^]^ Notably, KEGG enrichment analysis revealed that RRM2 was involved in the activation of the PI3K‐AKT and PD‐L1/PD‐1 pathways; these two pathways overlapped in the TCGA‐KIRC dataset (**Figure** **6**A). Thus, we hypothesized that RRM2 promotes PD‐L1 expression via AKT signaling in RCC. To test this hypothesis, we silenced RRM2 in cells treated with or without MK2206 (Figure 6B). RRM2 silencing did not further decrease AKT phosphorylation or PD‐L1 levels in the MK2206 pretreatment group (Figure 6B). In contrast, RRM2 overexpression elevated AKT phosphorylation and PD‐L1 levels in MK2206‐pretreated renal cancer cells (Figure 6C). ANXA1 knockdown reduced the mRNA and protein levels of PD‐L1 in renal cancer cells, whereas ANXA1 overexpression had the opposite effect (Figure 6D–G). Furthermore, the protein and mRNA levels of ANXA1 were significantly associated with those of PD‐L1 in patients with renal cancer or other malignancies (Figure S3C–F, Supporting Information). MK2206 pretreatment attenuated PD‐L1 downregulation upon ANXA1 silencing in renal cancer cells (Figure 6H), suggesting that the ANXA1‐mediated increase in PD‐L1 expression is dependent on the AKT pathway. Similarly, ANXA1 knockdown inhibited the ability of RRM2 to regulate PD‐L1 expression levels in renal cancer cells (Figure 6I,J). Hence, we then examined the role of RRM2 in the efficacy of PD‐1 blockade in renal cancer in vivo. Consistent with our findings in human RCC cells, RRM2 silencing decreased ANXA1 levels, AKT phosphorylation levels, and PD‐L1 levels in murine RCC cells (Renca; Figure 6K). Additionally, RRM2 knockdown in Renca cells suppressed tumor growth in immunocompetent mice (Figure 6J–H). Importantly, RRM2 silencing enhanced the antitumor effects of PD‐1 blockade in immunocompetent mice by promoting the infiltration of CD45^+^CD8^+^ and inhibiting the infiltration of CD11b^+^Gr1^+^ myeloid cells in tumors (Figure 6L–O) (Figure 6K). Therefore, these results suggest that RRM2 inhibition may augment the antitumor efficacy of immune checkpoint blockade in patients with RCC.

## 3 Discussion

Inactivating mutations in *VHL* are common in ccRCC, and systemic therapy using TKIs has become the standard of care.^[^ ^25^ ^]^ However, drug resistance remains a major obstacle that limits the long‐term survival benefits of patients with RCC.^[^ ^26^ ^]^ Hence, exploring the mechanism of TKI resistance is required in order to identify novel targetable candidates. Apart from primary resistance, secondary (also known as acquired) resistance after initial tumor regression has been reported in patients with RCC.^[^ ^8^ ^]^ Alternative activation of the PI3K/AKT/mTOR signaling pathway after long‐term therapy is believed to play a critical role in TKI resistance in RCC by increasing HIF and VEGF levels and promoting angiogenesis.^[^ ^27^ ^]^ Factors promoting AKT pathway activation include interleukin‐6 (IL‐6)^[^ ^28^ ^]^ and interleukin‐8 (IL‐8).^[^ ^29^ ^]^ Treatment with IL‐6^[^ ^30^ ^]^ or IL‐8^[^ ^31^ ^]^ neutralizing antibodies was found to avoid TKI resistance in RCC.

ANXA1 has been shown to bind to the FPRs, FPR1 and FPR2, thereby activating the PI3K/AKT pathway in breast cancer.^[^ ^18^ ^]^ A human phosphokinase antibody array study indicated that ANXA1 activated AKT signaling and inhibited autophagy in nasopharyngeal carcinoma cells.^[^ ^20^ ^]^ Additionally, ANXA1 upregulation resulted in AKT inhibitors and trastuzumab resistance through activation of the HER2/PI3K/mTOR pathway.^[^ ^19^ ^]^ Hence, these findings suggest that ANXA1 activates the AKT pathway and may represent a promising therapeutic target to reverse drug resistance in cancer cells. We have demonstrated that UBE3A not only degraded ANXA1 in cervical carcinoma cell lines but was also involved in determining the stability of ANXA1 in renal cancer cells. Moreover, we found that RRM2 interacted with and stabilized ANXA1 in renal cancer cells by competing with UBE3A. We have also shown that the RRM2/ANXA1/AKT axis contributes to sunitinib resistance in RCC. In addition, from our data it can be noticed that knockdown of RRM2 failed to further decrease AKT S473 phosphorylation in renal cancer cells with co‐knockdown ANXA1. However, xenografts with co‐knockdown of RRM2 and ANXA1 grew slower than ANXA1 or RRM2 knockdown alone, although the difference was not statistically significant (Figure 3M,N). Hence, these data indicate that RRM2 independent role of ANXA1 may also be important for RCC tumor growth. Thus, the specific role of RRM2 in regulating RCC proliferation requires further study.

RRM2 is a ribonucleotide reductase component that synthesizes deoxyribonucleotides required for mitochondrial and nuclear DNA repair and replication.^[^ ^32^ ^]^ RRM2 is considered an oncogenic protein that is upregulated in various types of cancer.^[^ ^33^ ^]^ In this study, we have systemically investigated the tumor‐promoting roles of RRM2 in RCC. We found that RRM2 was upregulated in RCC tissues, especially in those from sunitinib‐resistant patients. Consistent with previous findings,^[^ ^34^ ^]^ RRM2 overexpression promoted tumor growth and sunitinib resistance in renal cancer by regulating hypoxia and AKT pathway activation. Though it has been reported in previous studies that RRM2 activates the AKT pathway in cancer cells,^[^ ^35^ ^]^ the underlying mechanisms have not been identified. In our study, we found that RRM2 activated AKT by increasing the protein levels of ANXA1, independent of the enzymatic activity of RRM2. A major shortcoming of this study is that pretreatment with RRM2 inhibitors (COH29) or transfection with K95Q mutant RRM2 could not completely exclude the effect of RNR function.

Clinical studies have shown that PD‐1/PD‐L1 inhibitors are effective in 30–40% of patients with mRCC.^[^ ^36^ ^]^ PD‐1/PD‐L1 inhibitors, in combination with TKIs, significantly prolong the survival of patients with RCC.^[^ ^37^ ^]^ Understanding the mechanisms that regulate PD‐L1 expression in RCC may improve the therapeutic efficacy of PD‐1/PD‐L1 blockade. We have shown that RRM2 is closely associated with antitumor immune responses and that the RRM2/ANXA1/AKT axis promotes PD‐L1 expression in renal cancer cells. RRM2 inhibitors are divided into two types:^[^ ^38^ ^]^ drugs that inactivate the function of RNR (e.g., hydroxyurea^[^ ^39^ ^]^ and COH29^[^ ^40^ ^]^) and those that downregulated RRM2 (RRM2 antisense oligonucleotide GTI2040^[^ ^41^ ^]^ and the siRNAs CALAA‐01^[^ ^42^ ^]^). Our results suggest that RRM2 stabilizes ANXA1 independent of its RNR activity. Phase II clinical trials have shown that GTI2040 has a poor antitumor effect in patients with RCC^[^ ^43^ ^]^ because of poor organ specificity. Some of the limitations of GTI2040 can be avoided in CALAA‐01 by encapsulating RRM2‐specific siRNA within nanoparticles, and its efficacy has been evaluated in phase I clinical trials in patients with melanoma;^[^ ^42^ ^]^ however, sufficient evidence for the antitumor effects of CALAA‐01 is lacking. Thus, it is imperative to develop new RRM2 inhibitors for RCC treatment.

**Figure 7:**
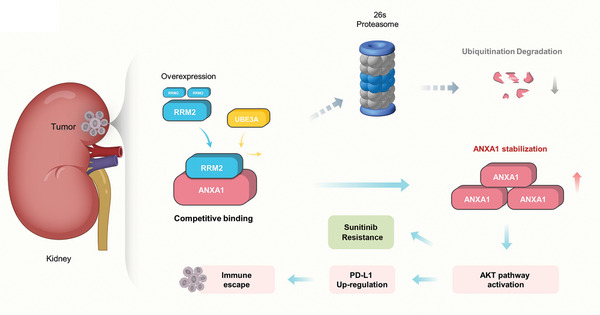
A hypothetical model depicting that the overexpressed RRM2 competed with UBE3A to prevent ANXA1 degradation. The RRM2‐ANXA1‐AKT axis regulated the sensitivity of sunitinib and PD‐1 blockade in renal cancer.

In conclusion, our results suggest that RRM2 overexpression in renal cancer cells plays a key role in sunitinib resistance in patients with RCC and that RRM2 competes with UBE3A to bind to the C‐terminus of ANXA1, preventing ANXA1 degradation. We have also provided evidence that the RRM2‐ANXA1‐AKT axis regulates sensitivity to sunitinib and PD‐1 blockade in renal cancer cells (**Figure** **7**). Therefore, RRM2 inhibition may enhance the antitumor effects of PD‐1/PD‐L1 inhibitors in combination with TKIs.

## 4 Experimental Section

### Cells Lines, Cell Culture, and Chemical Agents

The human renal cancer cell lines 786‐O (CL‐0010) and A498 (CL‐0254), and the murine renal cancer cell line Renca (CL‐0568) were purchased from Procell Life Science&Technology (Wuhan, China). All cells were subjected to STR authentication in Procell Life Science&Technology. 786‐O cells were cultured with RPMI‐1640 (PM150110, Procell Life Science&Technology) supplemented with 10% Fetal Bovine Serum (FBS) (164210‐500, Procell Life Science&Technology) and 1% Penicillin‐Streptomycin (P/S) (PB180120, Procell Life Science&Technology). A498 cells were maintained in MEM (PM150410, Procell Life Science&Technology) supplemented with 10% FBS (164210‐500, Procell Life Science&Technology) and 1% P/S (PB180120, Procell Life Science&Technology). Renca cells were cultured in a specific culture medium provided by Procell Life Science&Technology (CM‐0568). All the mentioned cells were maintained in 5% CO2 at 37 °C.

To construct sunitinib‐resistant 786‐O cells (786‐O R), we followed a previously reported protocol.^[^ ^15^ ^]^ The RPMI‐1640 medium was supplemented with 40 µm sunitinib (S7781, Selleck, China) for 2 weeks. The cells were then cultured with RPMI‐1640 without sunitinib for another 2 weeks. Then, the cells were cultured with RPMI‐1640 with 40 µm sunitinib for 2 weeks and RPMI‐1640 without sunitinib for another 2 weeks. This process was repeated to four times.

RRM2 inhibitors (COH29, S0283), MG132 (S2619), and cycloheximide (CHX, S7418) were obtained from Selleck.

### Cell Transfection and Lentivirus Based RNA Interference

Plasmids RRM2, UBE3A, and ANXA1 were obtained from GENECHEM (Shanghai, China) and WZ bioscience (China). The KOD‐Plus‐Mutagenesis Kit (Cat #SMK‐101B, TOYOBO, Japan) was used to synthesize the RRM2‐K95Q mutant. Lipofectamine 2000 (Thermo Fisher Scientific, Shanghai, China) was used to transfect the plasmids. Lentivirus‐based short hairpin RNAs (shRNAs) were purchased from Sigma (USA). For RNA interference, gene‐specific shRNA in combination with pVSVG and pEQXV were transfected into 293T cells. Twenty‐four hours post transfection, the culture medium was replaced with fresh culture medium with 10% FBS. After 24 h, the medium without 293T cells was harvested and mixed with the medium containing cultured renal cancer cells. Forty‐eight hours later, puromycin (10 µg mL^−1^) was used to select positive renal cancer cells infected with shRNAs.

### Statistical Analysis

The experimental data are presented as mean ± standard deviation (mean ± SD). The sample size (*n*) for each statistical analysis is provided in the figure legends. GraphPad Prism 5 software was used to calculate the *p* value using the unpaired two‐sided Student's *t* test for comparison of difference between two groups or one‐way analysis of variance (ANOVA) followed by Turkey's multiple comparisons post hoc test for comparison of differences between more than two groups. Differences were considered statistically significant at *p* values less than 0.05. In all cases, statistical differences were considered at *, *p* < 0.05; **, *p* < 0.01; ***, *p* < 0.001; not significant (ns), *p* > 0.05.

Other methods used for this study are provided in the Supporting Information.

## Conflict of Interest

The authors declare no conflict of interest.

## Author Contributions

X.J.: funding acquisition, investigation, methodology, project administration, writing – original draft; W.X.: methodology, writing – original draft; B.Z.: software, formal analysis, methodology; H.Y.: software, methodology; L.Z.: conceptualization; L.Y.: project administration, writing – original draft, writing – review & editing.

## Supporting information

Supporting Information

## Data Availability

The datasets used and/or analyzed during the current study are available from the corresponding authors (Xin Jin, jinxinxy2@csu.edu.cn) on reasonable request.
