# Supplementary material for: RRM2 Regulates Sensitivity to Sunitinib and PD‐1 Blockade in Renal Cancer by Stabilizing ANXA1 and Activating the AKT Pathway
Source: Adv Sci (Weinh). 2021 Jul 28;8(18):2100881. doi: 10.1002/advs.202100881 (PMC8456228; doi:10.1002/advs.202100881)
Supplement: Supplementary file 1 — Supporting Information [file ADVS-8-2100881-s001.pdf]

© 2021 Wiley-VCH GmbH

ADVANCED SCIENCE
Open Access

Supporting Information

for *Adv. Sci.,* DOI: 10.1002/advs.202100881

RRM2 regulates sensitivity to sunitinib and PD-1 blockade in renal cancer by stabilizing ANXA1 and activating the AKT pathway

Wei Xiong, Bin Zhang, Haixin Yu, Liang Zhu, Lu Yi, Xin Jin

RRM2 regulates sensitivity to sunitinib and PD-1 blockade in renal cancer by stabilizing ANXA1 and activating the AKT pathway

Wei Xiong, Bin Zhang, Haixin Yu, Liang Zhu, Lu Yi, Xin Jin

Supplementary methods and material

Co-immunoprecipitation and immunoblotting

Ethical approval for the use of human tissues (Renal cancer patients with or without sunitinib resistance) was obtained by the local ethics committee (The Second Xiangya hospital, China) (Approval No. 2021068). Written informed consent was acquired from all patients before surgery. For co-immunoprecipitation, cells were harvested and incubated in 1mL of RIPA buffer for 20 min on ice. Cell lysates were centrifuged at 12000 rpm for 15 min at 4ºC. The supernatant after centrifugation was collected and incubated with Pierce Protein G Agarose (Thermo Fisher Scientific, USA) and primary antibody or IgG at 4ºC overnight. The beads were washed 5 times with RIPA buffer, resuspended with loading buffer, and boiled at 100ºC for 5 min. The supernatant was subjected to immunoblotting. Pancreatic cancer whole-cell lysates were obtained in RIPA buffer, freshly supplemented with 1 mM phenylmethanesulfonyl fluoride (PMSF). Protein concentration was assessed by the BCA method. Equal amounts of protein were resolved by SDS-PAGE and transferred onto PVDF membranes. Subsequently, membranes were incubated with primary antibodies for more than 8 hours at 4ºC. Next, membranes were probed with the appropriate secondary antibody for 1 hour at room temperature. Signal intensities were measured using the Chemiluminescent Western Blot Detection Kit (cat. no. 32209, Thermo Fisher Scientific, USA). The following primary antibodies were used in our study: RRM2 (11661-1-AP, proteintech;

1:2000 dilution), GAPDH (ab8245, Abcam; 1:5000 dilution), ANXA1 (21990-1-AP, proteintech; 1:10000 dilution), UBE3A (10344-1-AP, proteintech; 1:1000 dilution), pAKT-S473 (66444-1-Ig, proteintech; 1:500 dilution), pAKT-S473 (4056S, CST Cell Signaling Technology, 1:200 dilution), AKT (10176-2-AP, proteintech; 1:2000 dilution), PD-L1 (66248-1-Ig，proteintech; 1:1000 dilution) and Beta-actin (66009-1-Ig, proteintech; 1:4000 dilution) The proteasome inhibitor MG-132 (Cat. No. S2619) was purchased from Selleckchem.

Quantitative real-time PCR (qRT-PCR)

Total RNA from pancreatic cancer cells was extracted using TRIzol (Thermo Fisher Scientific, USA). Reverse transcription was conducted to generate cDNA (PrimeScript™ RT reagent Kit). qRT-PCR analysis was carried out using TB Green™ Fast qPCR Mix. Relative mRNA levels of target genes were calculated using the $2^{-\Delta\Delta}$Cq method after normalization to GAPDH mRNA levels. The sequences of the forward and reverse primers are provided in Supplementary Table S2.

MTS assay

Renal cancer cells (1 × 105) were seeded in 96-well plates, and MTS reagent was added according to the manufacturer's protocol (cat. no. ab197010, Abcam). Absorbance at 490 nm was measured to evaluate in vitro cell growth. For colony formation assay，the cells were seeded into 6-well plates (500 cells/well) and incubated in prescribed medium with 10% FBS at 37°C.

Mice study

All animal procedures were approved by the Ethics Committee of Xiangya Medical College, Central South University (Approval No. 2021095). BALB/c-nude mice (4-5 weeks old, 18-20 g) were purchased from Shulaobao Biotech (Wuhan, China). 786-O cells were infected with the

indicated lentiviral particles. After puromycin selection for 72 hours, cells (1×10^7 per mouse) were subcutaneously injected into the back of mice. The length and width of xenografts were measured using a Vernier caliper, and tumor volumes were calculated using the formula $(L \times W^2)/2$. When tumor volume reached 100mm^3, randomly picked (by using a random number table) tumor-bearing mice were treated with sunitinib (20mg/kg) by oral gavage every other day. After mice were euthanized, tumors were excised and weighted.

Six-week-old C57BL/6 mice were purchased from Shulaobao Biotech (Wuhan, China). Renca cells (1×10^7 in 100 µl 1×PBS) infected with shControl or shRRM2 lentivirus were injected s.c. into the right flank of mice. After the xenografts reached a size of approximately 50 mm^3, mice carrying similar types of tumors were randomized into different groups and treated with anti-PD-1 (BioXcell, Clone RMP1-14)/IgG (BioXcell, Clone 2A3) (200 µg, i.p., given at days 0, 3, 6). Mice were euthanized and tumors were collected from all animals once the tumors reached a volume of 1000 mm^3.

Flow cytometry analysis

The 786-O cells were harvested and washed with PBS. Cells were fixed in 4% paraformaldehyde for 15 min. After washing with PBS, cells were incubated with ice-cold 100% methanol for 30 min on ice. Cells were washed with PBS and incubated with PD-L1 antibody (Biolegend, APC anti-human CD274, clone 29E.2A3) or isotype IgG (Biolegend,APC anti-human IgG Fc Antibody, clone HP6017) for 15 min at room temperature. After washing three times with PBS, the cells were resuspended in PBS and analyzed by flow cytometry.

For flow cytometry analysis of the mouse tissue samples, the tumors were cut into small pieces and digested with 2 mg/ml collagenase (Sigma, USA) in DMEM for 1 h at 37°C. Cells were

filtered through a 70 μm nylon strainer and resuspended in red blood cell lysis buffer (Biolegend) for 3 min at room temperature. The cells were then suspended in PBS with 2% BSA and costained with the following antibodies: CD45 (Biolegend, 103112, APC conjugated); CD8 (Biolegend, 100708, PE conjugated); CD11b (Biolegend ,101212, APC conjugated); and Gr1 ((Biolegend, 108406, FITC conjugated)). After incubation with antibody for 15 min, the cells were washed with PBS and analyzed by flow cytometry.

Tissue microarray and immunohistochemistry (IHC)

The tissue microarray slides were purchased from Avilabio (Xi'an, China) (KD1921). The tissue microarray specimens were immunostained with PD-L1 (Cell Signaling Technology, 13684, dilution 1: 1000), RRM2 (11661-1-AP, proteintech; 1:1000 dilution) and ANXA1 (21990-1-AP, proteintech; 1:10000 dilution). Staining intensity was scored in a blinded fashion: 1 = weak staining at 100× magnification but little or no staining at 40× magnification; 2 = medium staining at 40× magnification; 3 = strong staining at 40× magnification. The degree of immunostaining was reviewed and scored by two independent pathologists who were blinded to the clinical details. The scores were determined by the percentage of positive cells multiplied by the staining intensity.

Supplementary Figure 1

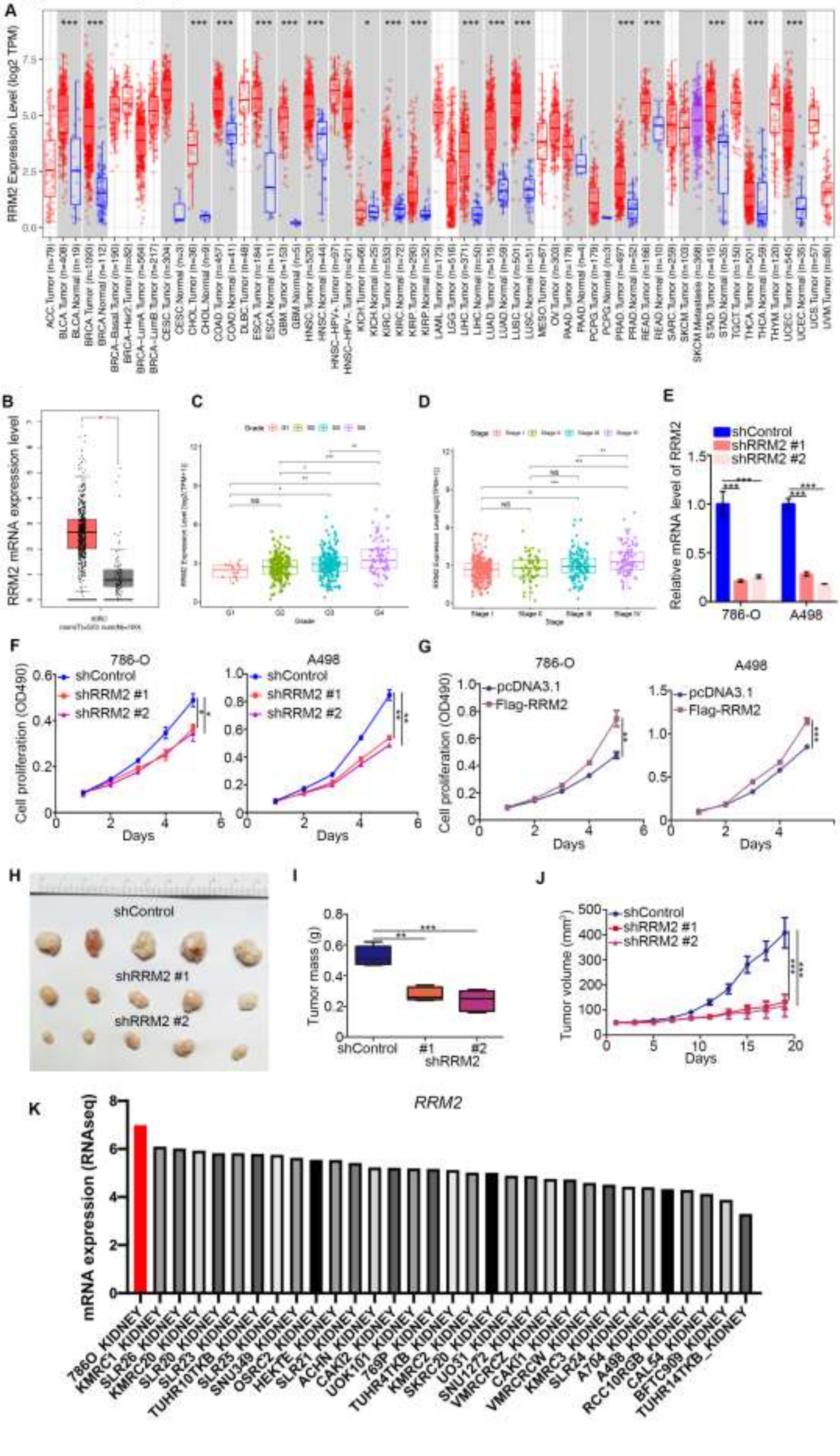

Supplementary Figure 1. A, Analysis the mRNA expression level of RRM2 in various types of cancer as indicated. **B**, the mRNA expression level of RRM2 in renal cancer and non-tumor renal tissues via analyzing the GEPIA web tool. **C and D**, the expression levels of RRM2 in different grades (C) or stages (D) of renal cancer patients. P values as indicated. **E and F**, 786-O and A498 cells were infected with lentivirus expressed shControl or shRRM2. After 72 h infection and puromycin selection, cells were harvested for RT-qPCR and MTS assay. Data presented as Mean ± SD with three replicates. *, $P < 0.05$; **, $P < 0.01$; ***, $P < 0.001$. **G**, 786-O and A498 cells were transfected with indicated plasmids. After 24 h, cells harvested for RT-qPCR assay. Data presented as Mean ± SD with three replicates. **, $P < 0.01$; ***, $P < 0.001$. **H-J**, 786-O cells were infected with lentivirus expressed shControl or shRRM2. After 72 h infection and puromycin selection, cells were subcutaneously injected into nude mice for xenografts assay. The image of tumor was shown in panel H. The tumor mass was demonstrated in panel I. The tumor growth curve was indicated in panel J. Data presented as Mean ± SD with five replicates. One-way ANOVA followed Turkey's multiple comparisons post hoc test was applied for the statistical analysis. **, $P < 0.01$; ***, $P < 0.001$. **K,** the RRM2 mRNA expression in different renal cancer cell lines were analyzed by the Cancer Cell Line Encyclopedia (CCLE) [http://www.broadinstitute.org/ccle] datasets.

Supplementary Figure 2

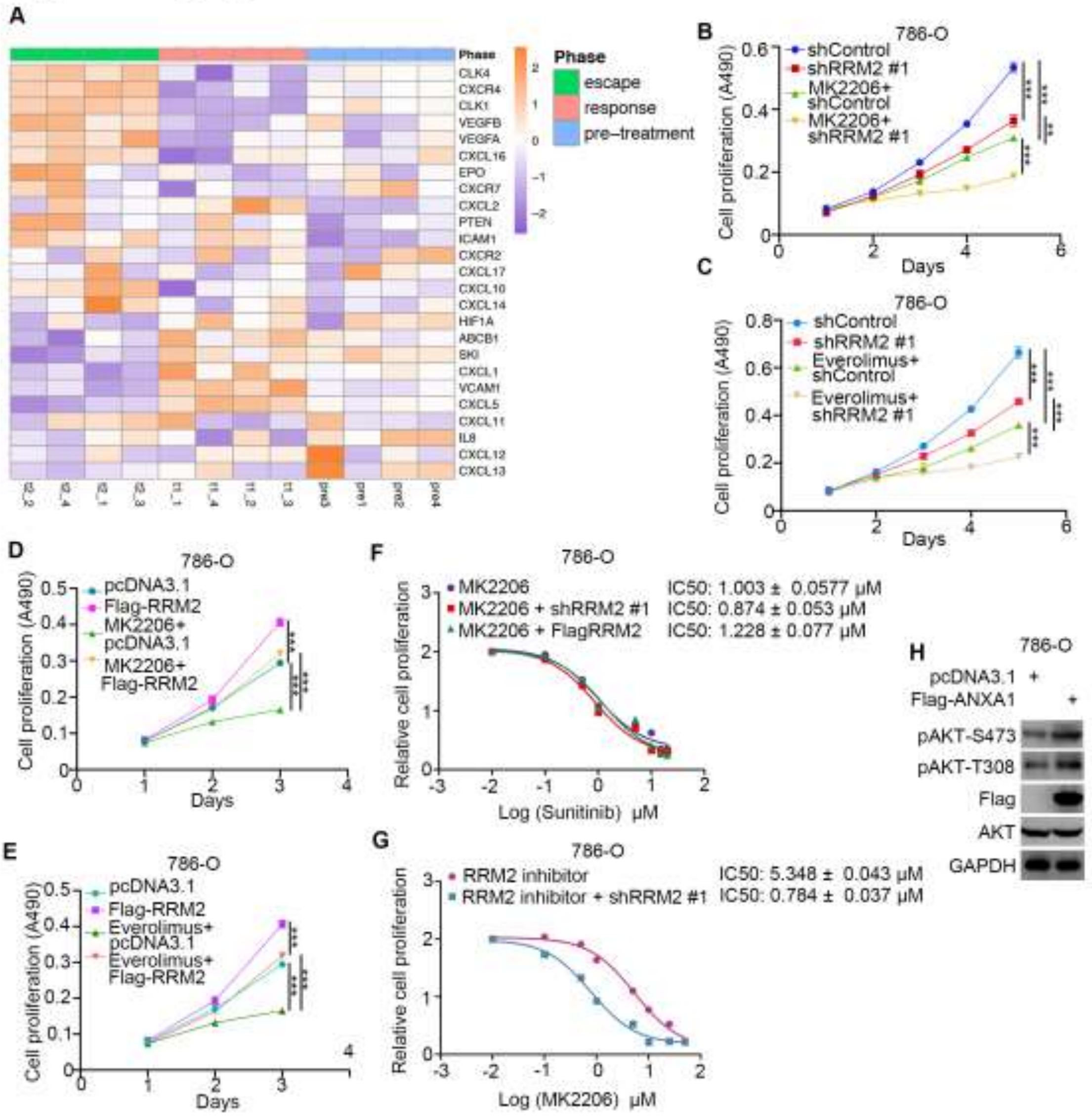

Supplementary Figure 2. A, Hierarchical clustering and heat map analysis of top 25 genes involved in sunitinib response and resistance (escape) in GSE76068. **B,** 786-O cells were infected with indicated shRNAs for 72 h. Cells were treated with or without 30 µM MK2206 for 24 h. Cells were harvested for MTS assay. Data presented as Mean ± SD with three replicates. One-way ANOVA followed Turkey’s multiple comparisons post hoc test was applied for the statistical analysis. **, P < 0.01; ***, P < 0.001. **C**, 786-O cells were infected with indicated shRNAs for 72 h. Cells were treated with or without 50 µg Everolimus for 24 h. Cells were harvested for MTS assay. Data presented as Mean ± SD with three replicates. One-way ANOVA followed Turkey’s multiple comparisons post hoc test was applied for the statistical analysis. ***, P < 0.001. **D**,

786-O cells were transfected with indicated shRNAs for 24 h. Cells were treated with or without 30 μM MK2206 for 24 h. Cells were harvested for MTS assay. Data presented as Mean ± SD with three replicates. One-way ANOVA followed Turkey's multiple comparisons post hoc test was applied for the statistical analysis. ***, P < 0.001. **E**, 786-O cells were infected with indicated shRNAs for 24 h. Cells were treated with or without 50 μg Everolimus for 24 h. Cells were harvested for MTS assay. Data presented as Mean ± SD with three replicates. One-way ANOVA followed Turkey's multiple comparisons post hoc test was applied for the statistical analysis. ***, P < 0.001. **F**, 786-O cells were pretreated with 20 μM MK2206. These cells were infected with shControl or shRRM2 for 72 h. Then shRRM2 group cells were transfected with or without RRM2 plasmids for 24 h. Cells were treated with a series of concentration of sunitinib for 24h. Cell viability was measured by MTS assay. **G**, 786-O cells were pretreated with 20 μM COH29. These cells were infected with shControl or shRRM2 for 72 h. Cells were treated with a series of concentration of MK2206 for 24h. Cell viability was measured by MTS assay. **H**, 786-O cells were transfected with indicated plasmids for 24 h. Cells were harvested for the Western blotting analysis.

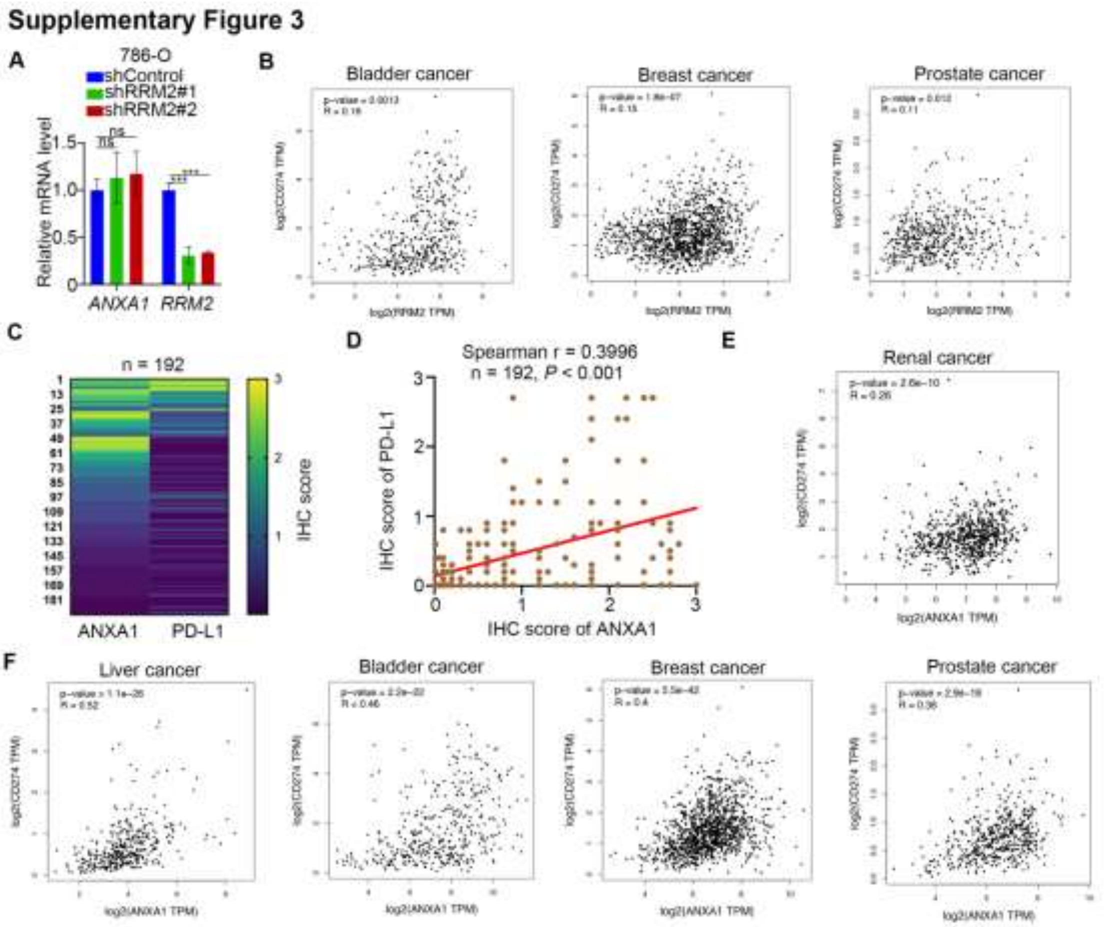

Supplementary Figure 3. A, 786-O cells were infected with shControl or shRRM2 for 72 h. Cells were harvested for Rt-qPCR assay. Data presented as Mean ± SD with three replicates. One-way ANOVA followed Turkey's multiple comparisons post hoc test was applied for the statistical analysis. Ns, not significant; ***, P < 0.001. **B**, the relationship between the mRNA levels of RRM2 and CD274 in bladder, breast and prostate cancer were analyzed by the GEPIA web tool. **C and D**, the protein expression level (C) and correlation (D) of ANXA1 and PD-L1 from the TMA as shown. **E**, the relationship between the mRNA level of RRM2 and CD274 in renal cancer was analyzed by the GEPIA web tool. **F**, the relationship between the mRNA levels of RRM2 and CD274 in liver, bladder, breast and prostate cancer were analyzed by the GEPIA web tool.

Table S1: Sequences of RT-qPCR primers

**Species**	**Gene**	**Forward (5’-3’)**	**Reverse (5’-3’)**
Human	*GAPDH*	CCAGAACATCATCCCTGCCT	CCTGCTTCACCACCTTCTTG
Human	*RRM2*	ACAGAAGCCCGCTGTTTCTA	CCCAGTCTGCCTTCTTCTTG
Human	*ANXA1*	GCAGGCCTGGTTTATTGAAA	GCTGTGCATTGTTTCGCTTA
Human	*PD-L1*	GGTGCCGACTACAAGCGAAT	AGCCCTCAGCCTGACATGTC

Table S2: Sequences of gene-specific shRNAs

shRRM2-1	5′- CCGGGCAGACAGACTTATGCTGGAACTCGAGTTCCAGCATAAGTCTGTCTGCTTTTT[illegible] -3′
shRRM2-2	5′- CCGGCCCATTTGACTTTATGGAGAACTCGAGTTCTCCATAAAGTCAAATGGGTTTTTG -3′
shANXA1-1	5′- CCGGGCATTCTATCAGAAGATGTATCTCGAGATACATCTTCTGATAGAATGCTTTTT[illegible] -3′
shANXA1-2	5′- CCGGGCCTTGTATGAAGCAGGAGAACTCGAGTTCTCCTGCTTCATACAAGGCTTTTT[illegible] -3′

Table S3. Mass spectrometry of RRM2

Accession	Gene	Mw(kDa)	RRM2-IgG	RRM2-IP	log2(RRM2-IP/RRM2-IgG)	Diff Sig
P04083	ANXA1	38.71	19.2299	29.2485	10.019	++

P31350	RRM2	44.88	21.0895	31.0046	9.9151	++
P09874	PARP1	113.1	22.8697	31.4702	8.6005	++
H3BT71	RBMX	32.2	18.2314	25.7414	7.51	++
P61626	LYZ	16.54	20.4988	27.858	7.3592	++
D6RD46	LIMCH1	109.9	19.4776	26.2131	6.7355	++
P28799	GRN	63.54	19.2114	24.7511	5.5397	++
A0A0U1RR97	DOCK7	11.77	20.0519	26.3751	6.3232	++
P22626	HNRNPA2B1	37.43	21.5638	27.8402	6.2764	++
P47914	RPL29	17.75	25.23	26.2516	1.0216	+
P16401	HIST1H1B	22.58	19.8246	26.0597	6.2351	++
A0A0D9SFK2	MYO18A	231.1	19.5277	25.6333	6.1056	++
P83731	RPL24	17.78	19.8612	25.8369	5.9757	++
P22061	PCMT1	24.64	19.2579	25.0885	5.8306	++
P42677	RPS27	9.461	19.6022	25.1803	5.5781	++
Q13404	UBE2V1	16.5	19.9128	21.3946	1.4818	+
P62273	RPS29	6.677	18.9954	24.3353	5.3399	++
Q14192	FHL2	32.19	20.3365	23.9362	3.5997	+
Q12906	ILF3	95.34	19.568	24.8142	5.2462	++
R4GN19	RPL36A	2.932	19.7158	24.9444	5.2286	++
D6RD47	RPS23	14.84	19.3896	24.5214	5.1318	++
A0A2R8Y5N1	DIAPH1	139.4	19.5452	24.6531	5.1079	++
O43707	ACTN4	104.9	20.7635	25.7273	4.9638	++
B8ZZK4	RPL31	8.988	22.1194	27.0806	4.9612	++
E5RJH3	RPL30	6.263	18.0911	23.0521	4.961	++
P67809	YBX1	35.92	18.8599	23.7652	4.9053	++
P60903	S100A10	11.2	19.2573	24.1484	4.8911	++
Q8IV04	TBC1D10C	49.71	20.75	25.5821	4.8321	++
O43390	HNRNPR	70.94	19.1859	24.0092	4.8233	++
P07476	IVL	68.48	19.4519	24.2663	4.8144	++
Q08188	TGM3	76.63	19.3654	24.0651	4.6997	++
Q14527	HLTF	113.9	19.6054	24.247	4.6416	++
Q7LG56	RRM2B	40.74	19.7774	24.4054	4.628	++
C9JG08	C2orf16	598.5	19.7094	24.3049	4.5955	++
F5H8D7	XRCC1	66.01	21.6724	26.2552	4.5828	++
B1AHC9	XRCC6	64.28	18.722	23.1703	4.4483	++
P35579	MYH9	226.5	27.2579	31.5314	4.2735	+
A0A2U3TZL8	KIF23	92.68	19.9823	24.191	4.2087	+
C9K0U8	SSBP1	14.13	20.2716	24.4726	4.201	+
P49916	LIG3	112.9	20.8304	24.9474	4.117	+
Q9H0H5	RACGAP1	71.03	19.736	23.849	4.113	+

P05109	S100A8	10.83	20.3059	24.4166	4.1107	+
P17096	HMGA1	11.68	19.2274	23.2919	4.0645	+
A8MXP9	MATR3	99.97	22.6826	24.5907	1.9081	+
Q8TD55	PLEKHO2	53.35	19.0778	21.6134	2.5356	+
O60437	PPL	204.7	21.1903	25.0653	3.875	+
J3QQM1	PSMC5	29.35	19.9505	22.5193	2.5688	+
P20700	LMNB1	66.41	21.1914	23.8932	2.7018	+
P51991	HNRNPA3	39.59	21.6952	25.4134	3.7182	+
P27694	RPA1	68.14	19.7983	23.4416	3.6433	+
Q92522	H1FX	22.49	19.1507	22.755	3.6043	+
Q9Y446	PKP3	87.08	19.2551	22.784	3.5289	+
F8W1R7	MYL6	16.29	23.503	27.0289	3.5259	+
P68871	HBB	16	18.5802	21.9927	3.4125	+
M0R3F1	HNRNPUL1	71.61	19.4761	22.8443	3.3682	+
Q9UN86	G3BP2	54.12	19.8105	23.175	3.3645	+
B1AHB1	MCM5	77.59	20.8259	24.1707	3.3448	+
A0A0C4DGG9	CHD4	220.5	19.5589	22.8976	3.3387	+
K7EMH1	RPL22	10.42	23.6125	25.2631	1.6506	+
B9A067	IMMT	78.97	20.0161	21.3927	1.3766	+
O43242	PSMD3	60.98	19.376	22.6805	3.3045	+
A8MX94	GSTP1	19.48	18.7633	22.0639	3.3006	+
Q9P107	GMIP	106.7	19.6892	22.9798	3.2906	+
Q9UBG3	CRNN	53.53	18.8766	22.1622	3.2856	+
P28288	ABCD3	75.48	20.1645	23.4437	3.2792	+
Q01081	U2AF1	27.87	20.2778	23.5349	3.2571	+
Q9Y3Y2	CHTOP	26.4	19.1528	22.3588	3.206	+
D6R9P3	HNRNPAB	30.3	19.2799	22.4677	3.1878	+
G5E9R5	ACP1	8.971	19.1788	22.3421	3.1633	+
F8W6I7	HNRNPA1	33.16	24.4838	27.6334	3.1496	+
P16403	HIST1H1C	21.36	25.1638	27.4119	2.2481	+
P19105	MYL12A	19.79	20.3888	23.5225	3.1337	+
C9J052	TREX1	18.41	21.4301	24.5582	3.1281	+
P11940	PABPC1	70.67	24.3229	27.4245	3.1016	+
K7ERG4	SNRPD2	8.777	20.4629	23.3611	2.8982	+
Q9NSD9	FARSB	66.12	19.2602	22.354	3.0938	+
Q03169	TNFAIP2	72.66	18.7442	21.8258	3.0816	+
E7ERL0	NME1	15.26	19.2399	22.3197	3.0798	+
D6RD66	WDR1	26.8	20.2075	23.2713	3.0638	+
P62861	FAU	6.648	19.2745	22.3347	3.0602	+
P62318	SNRPD3	13.92	19.1652	22.219	3.0538	+
Q8N163	CCAR2	102.9	19.9835	23.0157	3.0322	+
Q13835	PKP1	82.86	19.8369	22.845	3.0081	+

C9J352	KPNA1	19.39	19.7199	22.6658	2.9459	+
P60468	SEC61B	9.974	18.7898	21.7318	2.942	+
P43686	PSMC4	47.37	22.3712	24.4879	2.1167	+
P08708	RPS17	15.55	23.0928	26.0041	2.9113	+
Q9Y2T3	GDA	51	20.0897	22.9823	2.8926	+
Q9NZM1	MYOF	234.7	20.1814	23.0573	2.8759	+
B4DY09	ILF2	38.91	18.9517	21.7952	2.8435	+
B1AR63	HK1	32.55	22.9025	23.9402	1.0377	+
Q5JR95	RPS8	21.88	24.6158	27.409	2.7932	+
P05976	MYL1	21.15	19.3763	22.1645	2.7882	+
H0Y2V1	MAP4	48.65	19.8862	22.6606	2.7744	+
Q9NR30	DDX21	87.34	23.1521	25.9	2.7479	+
P14324	FDPS	48.28	19.1433	21.8509	2.7076	+
F8W7S5	RRBP1	84.33	20.4035	23.0958	2.6923	+
P35637	FUS	53.43	19.143	21.8106	2.6676	+
P42166	TMPO	75.49	20.4233	23.0818	2.6585	+
P39748	FEN1	42.59	20.6024	23.2558	2.6534	+
C9JTK4	AP2M1	18.32	21.4883	23.032	1.5437	+
E5RG31	MCM4	65.73	20.4998	23.099	2.5992	+
F1T0I1	SEC16A	249.5	23.9045	25.949	2.0445	+
Q09666	AHNAK	629.1	22.7252	23.8776	1.1524	+
P39019	RPS19	16.06	23.9029	25.4715	1.5686	+
P63151	PPP2R2A	51.69	19.3978	21.9308	2.533	+
Q71UM5	RPS27L	9.477	19.9491	22.4721	2.523	+
Q92817	EVPL	231.6	20.5516	23.0725	2.5209	+
Q86V81	ALYREF	26.89	21.1342	23.6176	2.4834	+
Q6NZI2	CAVIN1	43.48	19.2719	21.7467	2.4748	+
P06702	S100A9	13.24	23.3211	25.7659	2.4448	+
B4DDN1	QARS	71.74	20.7558	23.1834	2.4276	+
Q53H96	PYCR3	28.66	19.1403	21.5643	2.424	+
C9J6P4	ZC3HAV1	114.1	20.2994	22.7024	2.403	+
Q12965	MYO1E	127.1	21.2741	22.62	1.3459	+
A0A0D9SFL2	PNKP	53.12	19.9053	22.2721	2.3668	+
P07910	HNRNPC	33.67	23.0555	25.3839	2.3284	+
P11387	TOP1	90.73	23.0507	25.3558	2.3051	+
P04080	CSTB	11.14	20.5028	22.7879	2.2851	+
A0A0D9SF54	SPTAN1	282.8	20.0415	22.2806	2.2391	+
P62424	RPL7A	30	25.0977	27.3283	2.2306	+
Q9NX63	CHCHD3	26.15	19.5173	21.7429	2.2256	+
P68366	TUBA4A	49.92	20.8846	22.8888	2.0042	+
E2PSN0	RBM26	19.01	20.4817	22.6779	2.1962	+
P31949	S100A11	11.74	22.3347	24.5065	2.1718	+
P26639	TARS	83.43	19.56	21.7274	2.1674	+

E9PMS6	LMO7	145.4	20.7894	22.9242	2.1348	+
Q9C0C2	TNKS1BP1	181.8	21.2733	23.3896	2.1163	+
P60866	RPS20	13.37	23.5289	25.6431	2.1142	+
C9JJT5	ATP5MF-PTCD1	5.915	19.2735	21.386	2.1125	+
P42766	RPL35	14.55	23.9728	26.0834	2.1106	+
A0A087WUZ3	SPTBN1	274.8	21.064	22.3906	1.3266	+
O95816	BAG2	23.77	20.5683	22.6615	2.0932	+
E9PJD9	RPL27A	10.13	23.8606	25.4518	1.5912	+
P18124	RPL7	29.23	25.7316	27.2314	1.4998	+
Q96PK6	RBM14	69.49	21.6387	23.5485	1.9098	+
P62081	RPS7	22.13	24.1494	26.1901	2.0407	+
I3L3W9	ALDH3A1	8.687	20.2186	22.245	2.0264	+
Q5TEE2	HDAC1	24.54	18.7701	20.7961	2.026	+
H3BRG4	UQCRC2	44.63	19.5896	21.6003	2.0107	+
P33993	MCM7	81.31	23.2077	25.1968	1.9891	+
P62701	RPS4X	29.6	27.0189	28.1916	1.1727	+
A0A1X7SBS1	HNRNPU	81.79	26.3493	28.3057	1.9564	+
O14949	UQCRQ	9.906	19.2986	21.2295	1.9309	+
P31942	HNRNPH3	36.93	20.095	22.0259	1.9309	+
Q15654	TRIP6	50.29	20.8303	22.6584	1.8281	+
B0QZK9	HP1BP3	8.15	18.9674	20.469	1.5016	+
P04792	HSPB1	22.78	22.7317	23.9984	1.2667	+
P36578	RPL4	47.7	26.2518	27.6417	1.3899	+
A0A2R8YEM3	RPL15	19.97	25.9118	27.6849	1.7731	+
M0R1A7	RPL18A	17.48	25.9599	27.4658	1.5059	+
C9J1Z8	ARF5	17.11	20.1775	22.0289	1.8514	+
E7EPN9	PRRC2C	308.8	20.8958	22.7153	1.8195	+
P17655	CAPN2	79.99	20.8896	22.6834	1.7938	+
Q14134	TRIM29	65.83	22.6533	24.4121	1.7588	+
B4DXZ6	FXR1	68.33	19.9115	21.668	1.7565	+
Q3MHD2	LSM12	21.7	19.5187	21.2705	1.7518	+
P38646	HSPA9	73.68	28.5166	29.8991	1.3825	+
J3KTA4	DDX5	69.09	25.4048	27.1367	1.7319	+
P50990	CCT8	59.62	19.8818	21.547	1.6652	+
Q9HCY8	S100A14	11.66	19.2517	20.8972	1.6455	+
A0A494C0K9	ATP5PO	16.89	23.3414	24.4285	1.0871	+
P62750	RPL23A	17.7	24.8754	26.4801	1.6047	+
Q9UG63	ABCF2	71.29	20.3591	21.9602	1.6011	+
J3QRI7	RPL26	12.63	24.6061	26.1912	1.5851	+

E9PIY1	PACSIN3	37.21	18.5471	20.1318	1.5847	+
F8VU51	YLPM1	160.7	18.6911	20.2643	1.5732	+
D6RAN4	RPL9	20.78	23.1691	24.7009	1.5318	+
P62249	RPS16	16.45	25.4275	26.6999	1.2724	+
A0A087WXM6	RPL17	19.59	24.2581	25.825	1.5669	+
I3L0N3	NSF	82.09	20.4743	18.9174	-1.5569	-
G3V4X8	SNW1	43.33	19.4939	21.0481	1.5542	+
E9PP21	CSRP1	16.94	18.5382	20.0612	1.523	+
K7EIG1	CLUH	140.5	20.1441	21.6236	1.4795	+
A0A0B4J1Z1	SRSF7	15.76	18.6424	20.1085	1.4661	+
F5H6E2	MYO1C	119	19.3185	20.7561	1.4376	+
Q15717	ELAVL1	36.09	21.6868	23.1078	1.421	+
G3V203	RPL18	18.76	25.6014	26.9615	1.3601	+
P30041	PRDX6	25.04	20.4945	21.8915	1.397	+
P78559	MAP1A	305.5	20.8439	22.2392	1.3953	+
Q8J015	RPL13A	16.73	25.9649	27.3403	1.3754	+
A0A0D9SEM9	DDX39A	10.93	20.4008	21.7664	1.3656	+
D6RF44	HNRNPD	12.55	21.1252	22.4598	1.3346	+
P62277	RPS13	17.22	25.2444	26.2639	1.0195	+
E7EPB3	RPL14	14.56	23.5025	24.8302	1.3277	+
P27635	RPL10	24.6	26.2453	27.5233	1.278	+
D3YTB1	RPL32	15.62	24.2799	25.5503	1.2704	+
P02786	TFRC	84.87	22.1525	23.4103	1.2578	+
Q15424	SAFB	102.6	20.1551	21.3952	1.2401	+
Q8WUF5	PPP1R13L	89.09	20.3622	21.5935	1.2313	+
Q07666	KHDRBS1	48.23	24.1051	25.3256	1.2205	+
H9KV75	ACTN1	94.83	22.5148	23.7153	1.2005	+
Q8IVT2	MISP	75.36	19.4903	20.6895	1.1992	+
A0A024R4M0	RPS9	22.59	26.3499	27.4775	1.1276	+
P78344	EIF4G2	102.4	20.1027	21.2189	1.1162	+
P61353	RPL27	15.8	24.9099	25.9855	1.0756	+
Q86V48	LUZP1	120.3	20.4597	21.5153	1.0556	+
Q9BWF3	RBM4	40.31	20.1884	21.2372	1.0488	+
P61247	RPS3A	29.95	25.7623	26.8096	1.0473	+
Q60FE5	FLNA	278.2	20.2349	21.2671	1.0322	+
Q96D46	NMD3	57.6	19.6453	20.6654	1.0201	+
P15924	DSP	331.8	28.3482	29.3585	1.0103	+
Q16698	DECR1	36.07	19.9268	20.9343	1.0075	+
Q86U42	PABPN1	32.75	19.9656	20.9671	1.0015	+
